# The Organization of Diagnosis, Care and Funding for Specific Learning and Developmental Disorders (SLDD): A French Regional Experimental Protocol

**DOI:** 10.3389/fped.2021.652686

**Published:** 2022-01-05

**Authors:** Thiébaut-Noël Willig, Vincent Henry, Jean-Claude Netter, Patrick Contis, Cécile Castro-Gutierrez, Claire Oget-Gendre, Christophe Bonnier, Emilie Cabarrou, Laurent Raffier, Agnès Kabantchenko

**Affiliations:** ^1^Occitadys, Toulouse, France; ^2^Consultation de Pédiatrie, Clinique Ambroise Paré, ELSAN and EvEnTAil 31, Toulouse, France; ^3^Association Française de Pédiatrie Ambulatoire (AFPA), Orléans, France; ^4^SMPEA Peyre Plantade, Centre Hospitalier Universitaire (CHU) de Montpellier, Université de Montpellier, Montpellier, France; ^5^Centre de référence des troubles des apprentissages de Bigorre, Centre Hospitalier de Bigorre, Tarbes, France; ^6^Handicapped Children Institutes and Union Régionale des Professionnels de Santé Médecins Libéraux (URPS ML) Occitanie organisation, Toulouse, France; ^7^Centre Hospitalier de Carcassonne, Carcassonne, France; ^8^Équipe d'appui à la rapporteure générale ≪ Expérimentations innovantes en santé ≫, secrétariat général des ministères sociaux, ministère des solidarités et de la santé, Paris, France; ^9^Direction des Projets, Agence Régionale de Santé Occitanie, Montpellier, France; ^10^Société Coopérative et Participative (SCOP) Ipsofacto, Toulouse, France

**Keywords:** neurodevelopmental disorders, specific learning disorder (SLD), study protocol, health organization, medico economic evaluation

## Abstract

**Introduction:** Access in France to early diagnosis and care for the most severe, but infrequent, Neurodevelopmental Disorders (NDD), autism spectrum disorder and global developmental delay, in children aged 0–7 was improved through measures implemented in 2019. However, there are no such measures for specific learning disorders (SLD), attention, motricity and language disorders (SLDD), despite their annual incidence of between 5 and 8%.

**Method:** We describe the design of a new type of organization and financing of care for SLDD including evaluation procedure, as well as other factors, mainly at the prevention level that will contribute to local and national policy for this frequent health problem. This in response to a national call for projects, commonly called Article 51, targeted innovation in healthcare delivery and funding in the context of medium-term national reform. This provides project stakeholders with the opportunity to set up and implement “bottom-up” projects, mainly using local professionals. A joint initiative by the regional Health Authorities of the Occitanie region, the French Social Security system and a non-profit Association (Occitadys) proposed an experimental new structure of NDD care and funding.

**Discussion:** We here discuss the design of this experiment that aims, over two to three years, to alleviate families' financial burden of care and establish a regional three-tier care system with respect to evaluation, re-education and rehabilitation care. Our approach may benefit SLDD health-care planning, and addresses the questions of prevention, early detection and care-design for families, taking local and socioeconomic disparities into account.

## Introduction

Specific Learning Disorders (SLD), Specific Language Impairment (SLI), Developmental Coordination Disorders (DCD), and Attention Deficit Hyperactivity Disorder (ADHD) belong to an umbrella concept of Neurodevelopmental Disorders (NDD) that has become increasingly central since the publication of DSM5 (1). Health-care organization, however, depends on historical, epidemiological and regional or national factors that have to be considered to both better understand the current situation and design alternative models of planning and funding.

### History

In France, two different kinds of NDDhealth-care organizations have developed over time. The first, focusing on Autism Spectrum Disorders (ASD), was initially based on medico-social organizations that evolved to include evaluation and neuropsychological care under the aegis of the French Health Authority (*Haute Autorité de Santé*: HAS), with five consecutive generations of official recommendations and guidelines (2–6).

The second kind of organization covers specific developmental and learning disorders, including speech (SLI), motricity (DCD), attention (ADHD) and specific-learning disorders (SLD), which we here group together using the French acronym TSLA (*Troubles Spécifiques du Langage et des Apprentissages: SLDD*), as in a recent French national policy document (7). The social and medical care for these disorders can involve a number of different actors, including University hospitals, General hospitals, Rehabilitation structures, networks for deaf children, and professionals in private practice. A first step toward a coordinated response was the creation of a national network of reference centers in 2002, which were however quickly overwhelmed by the level of demand (8). In 2012, the CNNSE (*Commission Nationale de la Naissance et de la Santé de l'Enfant*) recommended a three-tier care structure. The first tier consists of school Doctors, general practitioners (GPs) and primary-care practitioners; the second was for complex disorders requiring multidisciplinary evaluation and care coordination; and the third was for patients with brain diseases, complex epilepsy, genetic disorders, and children who did not respond to standard rehabilitation processes, via reference centers (9). Over time, a number of coordinated care networks have been developed in various French regions, mainly based on local initiatives and often supported by Regional Health Agencies (*Agence Régionale de Santé*: ARS). These networks are also covered by a National Federation: the *Fédération Nationale des Réseaux de Santé Troubles du Neuro-développement et des Apprentissages de l'Enfant/Adolescent* (https://www.federeseauxdys.org/). In the meantime, National recommendations were issued by the HAS for ADHD, defining the health track to be followed at the first tier (10). Two national expert reports subsequently appeared, covering intellectual developmental disorders (11), and developmental coordination disorders (12).

Shortly afterwards, the HAS called for expertise to design national recommendations for specific language and learning disorders (including movement, speech and attention disorders), leading to a 2018 reference document: “*Comment améliorer le Parcours de Santé d'un enfant avec troubles spécifiques du langage et des apprentissages*?” (How to improve the health track for children with specific language or learning disorders) (7). Among the conclusions were the definition of the relationships between the prevention levels, including preschool and school, and the recourse to medical procedures if necessary, with a first tier for the screening and early identification of simple disorders, and second-line professionals and organizations for complex disorders. Evaluations and interventions were supposed to be based on common neurocognitive references, and second-tier organizations would rely on Medical Doctors with a special NDD qualification. A number of interventions were also suggested, but again not including any funding recommendations for political reasons. The ARS were expected to implement these recommendations, and in Occitanie this task was proposed to a regional non-profit association, Occitadys, which was created in June 2018, and chosen to address this priority as identified in the regional program for Health (PRS) 2018–2023 (13). The complete implementation of the 2018 recommendations was scaled down by the lack of funding for most of the diagnostic procedures and care. Only a certain fraction of the professionals involved (medical doctors, speech therapists and orthoptists) in France work under a national contract with Social Security, which defines their rules of practice and provides either direct payments to the professionals or reimbursement to patients. There is no such contract for psychomotricity specialists, occupational therapists or neuro-psychologists, who can either work in a public setting with their wages being paid from public funds, or in private practice (which is most often the case), in which case the whole financial burden falls on the patient.

### French Health Policy

On the political front, both Chambers of the French Parliament annually pass an Act defining the objectives and tools of the French Social Security system. The 2018 Act included a special section allowing for new innovation strategies with exemption from some of the usual Social-Security funding regulations. The goal was to experiment with modes of decision-making and local care-organization and funding, with the aim of applying successful projects at the national level (See Article 51 of the Social Security Financing Act for 2018: *Loi de Financement de la Sécurité Sociale*, LFSS) (14). The 2020 budget was 30M€, and the 64 pilots that were chosen in September 2020 had a total budget of €300 million over a five-year period. This resulted in a national plan produced by the French ministry of Health (MoH) and French Social Security under the auspices of the Strategic Council for Innovation in Health (*Conseil Stratégique de l'Innovation en Santé*: CSIS).

One year later, Article 62 of the 2019 LFSS defined the features of early interventions for ASD among NDD, with the subsequent creation of local centers for referral and early intervention, together with Social-Security funding for diagnoses and care for children under the age of seven with warning signs (*plateformes d'orientation et d'intervention précoce* TSA/TND) (15). The intention was to provide a rapid response to warning signs for children aged 0–7 (called PCO 0–7), mainly regarding ASD, but extended to families with children affected by other NDD. This did not, however, address children over age 7, with mostly SLD, DCD and ADHD problems.

### Occitanie Region

A five-year regional development plan (*Schéma Regional d'Organisation des Soins 2012-2017*) led to the organization of second-tier structures in the former Midi-Pyrénées region, but the lack of financial support meant that only seven of the nine French *départements* in the region were covered by either a second- or third-tier organization by the end of (13). An administrative reorganization in 2017 produced a larger region (Occitanie), with 13 *départements* and nearly 6 million inhabitants, and 60 000 births per year.

In January 2019, Occitadys was asked by ARS Occitanie, together with the local Social-Security representatives, to submit an application to establish a regional “*Parcours de santé TSLA*”, as defined by the HAS in 2018 (7). After 18-months work, the proposition was officially accepted in July 2020 for a 2–3 year period under the name “*Parcours de santé TSLA Occitanie*” (16), with the aim of a national roll-out in the case of success. In the meantime, the French President, Emmanuel Macron, announced during the National Handicap Conference on February 11^th^ 2020 the specific LD to be included in the priorities, as well as the measures to be extended up to child age 12, through the extension of the age range of age of the PCO, designated PCO 7–12. Our experimentation is thus being run in parallel with the implementation of the PCO 7–12 in other regions of France, and will extend the age range up to 15, fully include LDs, and finance all the steps of the second-level evaluations.

This article will describe how the experiment was constructed, the epidemiological background, the care structure proposed, the financial tools, and the evaluation process, as potential paradigms for both the design of experiments in other fields in France, and public-policy tools in other countries. The process described here represents in itself a research procedure applied to health organization.

## Methods/Design

### Local and National Background

The Occitanie region is located in the South West of France, with almost 6 million inhabitants, and the fastest annual population rise (+ 51.000), due to the global economic environment and its geographical location. It is also characterized by a high prevalence of financial precariousness (22% of those aged under 65) (17). The regional global-heath indicators appear good, but mask considerable disparities, partly due to geographical features (mountainous areas with restricted health services), two metropolitan areas (Toulouse and Montpellier), and a coastal area bordering the Mediterranean Sea that is characterized by poverty and unemployment.

Learning and developmental disorders have long-term consequences for those concerned, in terms of employment, social and economic integration, behavior, conduct and criminality. Poor literacy affects 9–12% of the French population, of whom up to one half have an underlying speech or reading disorder (18). This prevalence in Occitanie ranges from 8 to over 20% of young adults (19).

The delays in access to a qualified Doctor or a third-tier structure are between 3 to 18 months, with an accurate diagnosis being established on average by age 8 (20). However, local access varies by distance to the relevant establishment. The design of second-tier structures was initiated in the Western part of the region starting in 2012 and needs to be continued.

Most care for these disorders in France is supplied by the private sector, and majority of the cost is currently still not covered by Social Security. The average fee for specialized evaluation by a neuropsychologist is €250, and €150 for a psychomotricity specialist or an occupational therapist (21). Similarly, the reimbursement for Doctors involved in time-consuming evaluations is low, ranging from €28 up to €69.12 for complex visits lasting for up to one hour.

The structure of the process itself is also inadequate, as the first-line response for most children is a speech therapist, regardless of the condition; this is mostly (67%) at the parents' request, and/or after a teacher's alert (40%). Most of the first-line Doctors, who in France should prescribe evaluation by specialists such as occupational or speech therapists, have not received adequate training in the different fields of learning and developmental disorders. However, as a result of previous investment, 90% of children in Occitanie in 2019 were able to obtain second-tier expertise in either the private or public sectors (Occitadys, unpublished data). But prior to the experimentation, four of the thirteen *départements* in Occitanie did not yet have second-tier organizations, and families had to request evaluation in other areas, with long delays and distances, or forgo care: between 15 and 20% of families do end up forgoing care (21), while 30 to 40% of the others have to request Handicap status to qualify for financial support. This procedure has transferred costs from the health system to that dealing with compensation for disabilities (in France, this is the Departmental Handicap Office - *Maison Départementale des Personnes Handicapées*: MDPH), whose role is not to finance health.

Funding for public second-tier organizations has been an obstacle. Since these began to be introduced in 2012 in Occitanie, hospitals have had to apply to a special programme (“*Circulaire Frontière*”: an exemption to the tariff based on medical activity). This requires that all evaluations be made in one day at a hospital, with subsequent organizational constraints and the burden for the children of undergoing at least two neurocognitive evaluations on the same day.

Despite the efforts of universities and ongoing medical-education programs in the region, all of these drawbacks have led to a situation where the vast majority of MDs who have taken post-University training in learning disorders or NDD choose not to pursue clinical practice in this field.

### Epidemiology

Specific learning and developmental disorders share common epidemiological features: an incidence of 1 to 10%, as summarized in Table 1, with individuals affected by a single diagnosis being less frequent than situations of co-morbidity.

**Table 1:** Incidence and DSM codes for the main NDD involved in the project, as well as for differential diagnosis.

**Disorder DSM abbreviation – DSM code – (reference)**	**Incidence in the literature**
Developmental Coordination Disorder DCD - 315.4 - (22–25)	6%
Attention-Deficit / Hyperactivity Disorder ADHD - 314.00/01 - (26)	5.3%
Developmental Language Disorder DLD- 315.39 - (27–30) Severe DLD	1–7% 0.6 to 1%
Specific learning disorders with impairment in reading - 315.00 - (31, 32)	2.7 to 10%
Specific learning disorders with impairment in Mathematics - 315.1 - (33, 34)	3.6 to 6.5%
Other neurodevelopmental disorders not involved in the project	
Autism spectrum disorders ASD – 299 - (35–39)	1.7 to 7/1000
Intellectual developmental disorder IDD - 319 - (11, 40)	1.5 to 2%

The evaluation of the number of children needing a diagnostic procedure for complex NDD (apart from ASD and IDD) in Occitanie was extrapolated. In 2012–2017, we developed second-tier organizations in the Western part of the region, based on a mean incidence of 5% relative to the annual number of births.

To provide an updated evaluation of the number of children who may require treatment, we take the figure of 62 000 for the number of children aged 2 in 2016 in Occitanie (who will be 6 in 2020), and the national incidence rates from INSEE, producing a predicted number of 3720 children, of whom 3100 are in complex situations and 600 in simple situations (41) as detailed in Table 2.

**Table 2:** Lists the departmental distribution in Occitanie of simple situations (one disorder) and complex situations (two or more disorders, requiring multidisciplinary evaluation).

***Département*** **number and name**		**Live births**	**6% incidence rate (IR)**	**1% IR: simple situations**	**5% IR: complex situations**	**Annual number of children diagnosed complex cases**
09	Ariège	1 257	76	13	63	0
11	Aude	3 313	199	33	166	50
12	Aveyron	2 316	139	23	116	130
30	Gard	7 708	462	77	385	420
31	Haute-Garonne	16 024	961	160	801	714
32	Gers	1 383	83	14	69	18
34	Hérault	12 507	750	125	625	830
46	Lot	1 260	76	13	63	60
48	Lozère	595	36	6	30	0
65	Hautes-Pyrénées	1 907	114	19	95	150
66	Pyrénées-Orientales	4 429	265	44	221	248
81	Tarn	3 421	205	34	171	106
82	Tarn-et-Garonne	2 678	161	27	134	91
Whole region		58 798	**3528**	**588**	**2940**	**2871**

*INSEE – Number of births by département of the mother's residence in 2017 (41)*.

### Intervention Design

Occitadys was requested by ARS Occitanie and the local Social Security system to propose a re-organization of diagnosis and care, as well as Social-Security coverage for all of the expenses that were previously uncovered. This proposition was to be submitted to the Technical Committee for Innovation in Health (*Comité Technique de l'Innovation en Santé*: CTIS). A first draft was submitted as a letter of intent in April 2019, and Occitadys was chosen to undertake the project with the support of a key project enabler in July 2019. This step relied on a procedure involving the main partners of the project, through an attribution-ShareAlike international Creative Common license developed by the “Collectif co-design it” in 2015. (http://codesign-it-ventures.fr/approche-parkour/) (42). This stage was also supported by an expert from the inter-Ministerial delegation for ASD/NDD strategy. The second step was the specifications (*Cahier des Charges*), which Occitadys prepared with the help of an adviser, a worker cooperative (SCOP Ipso Facto). The first application in December 2019 was for a five-year experiment, revised to a 2 to 3-year experiment in May 2020 and accepted in June/July 2020.

The experiment was designed at three different levels: a core group, with the Occitadys Executive Committee and the external adviser in relation with ARS Occitanie, French Social Security, and the support team for Innovation Experimentation in Health (COG) of the MoH; a second group with the whole Occitadys Board; and a last group including external participants representing different types of regional organizations and research units.

In the meantime, and as an initial step for deployment, Occitadys worked with a Master of Public Health (MPH) intern in the Coordination of Health Tracks from the University Toulouse III-Paul Sabatier to update the reference guide for diagnostic tools (43) (from a first version of a regional guide from 2015), and define the role of the initial TSLA health-contact point (*correspondant d'entrée de parcours*).

### Operational Objectives

There were five operational objectives, including both the primary objectives of Occitadys and the specific objectives of the experiment.

Objective 1: **Structure of the first tier** (for those without diagnosis and questions regarding referral). This consisted of the design with the ARS of a map of the regional stock of Doctors and qualified paramedical/psychologists, listing Doctors who were already trained, organizing the training of new Doctors regarding disorders (with local Doctors' organizations), and establishing links to second-tier organizations. The experiment itself required an initial TSLA health-contact point to help families access the right professional or other relevant organizations, as well as informing first-tier Doctors about the experiment and how they could participate. This initial health-contact point had already been developed in the other care networks, but our experimentation will allow us to assess the strategies for the National scale-up.

**Figure 1:**
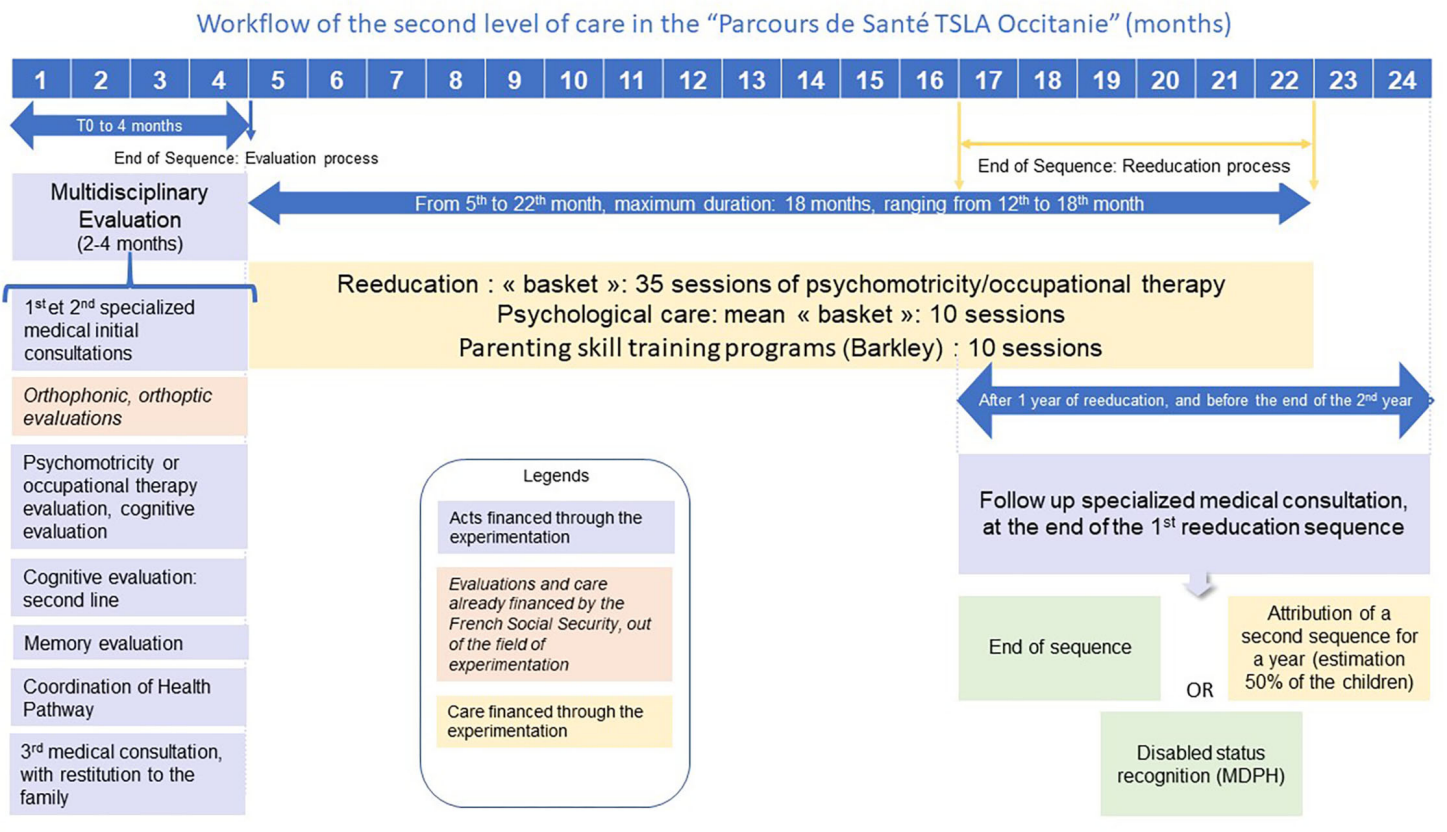
Shows the workflow diagram for the experimental test of second-tier care, as authorized.

Objective 2: **Test a new kind of funding based on a track approach**. The aim here was a smooth transition between the first and second tiers of diagnosis and care. For the first tier this included direct Social-Security funding of one year of psychomotricity/occupational-therapy rehabilitation (30 sessions), prescribed by the GP after the initial evaluation. For the second tier, patients/families were provided with access to a broad range of diagnosis and care with experimental direct Social-Security funding (details in Figure 1). This required all the medical evaluations to be carried out by a qualified NDD Doctor, who could then prescribe evaluations by psychometrician/occupational therapists, psychologist/neuropsychologists, and an evaluation of memory function, if needed. Coordination was also financed, including pluridisciplinary synthesis, and administrative and information-system costs. Other evaluations were also included: orthophonic assessments of speech, written language, mathematical cognition, and orthoptist evaluations may appear in the multidisciplinary summary, but were already funded directly by Social Security and were thus not affected by the new funding regime. The time-reduction goal is ideally a 2-4-month delay between the initial request for multidisciplinary assessment and the summary: this delay will be one of the items monitored by the external evaluator. Three types of care can be prescribed by the medical coordinator in charge of the child with direct Social Security funding: a year's rehabilitation by the psychometrician or occupational therapist (35 sessions); 10 psychology/neuropsychology sessions devoted to either the emotional consequences of the neurocognitive disorder or cognitive remediation; and 10 sessions of a training program for the child's parents, such as the Barkley program (44), in situations of impulsivity or opposition in ADHD. At the end of this first care sequence, a new formal evaluation by the medical specialist was planned to choose the next step: further rehabilitation, rehabilitation continued for a second year with funding in the experiment; care discontinued; or compensation requested through the MDPH.

Objective 3: **An integrated information system** shared between professionals, the respect of safety rules, and transfer of information and documents to the individual digital patient file. This information system will be used to determine the personalized plan for health coordination (PPCS), as defined in HAS recommendation of July 2019 (45) and will be the backbone for follow-up visits by telemedicine or tele expertise (as currently being implanted by the Social Security system).

Objective 4: **Improve the quality of interventions and local supply of TSLA**. This is mainly accomplished through the specific role of Occitadys. Along with the roll-out of the experiment, a regional reference document for diagnostic tools and procedures has been prepared by an expert group, to be validated by an independent professional group, and then endorsed by the Occitadys Board (43).

Objective 5: **Provide epidemiological data** on relevant disorders to support the national roll-out.

### Key Measures

We now describe some of the key features and measures implemented to attain these objectives.

#### First Tier: The Initial Contact Point

The goal was to organize the first response level via a local health-contact point. The role of this contact point is answer families' questions, with training provided to allow for a multidimensional analysis of needs through a simple flow process. This first level of care is aimed at children aged 6 to 15 with a presumed simple condition, i.e., with a complaint in only one field of development. This yields a first-line referral to either a GP, a second-tier organization, a regular school with adapted pedagogical measures, or more specialized resources such as rare-disease networks, ASD networks, or medico-social structures. There will be one part-time contact-point correspondent per *département*, with a budget based on the projected number of patients, administratively linked with a second-tier organization and potentially with other care providers. Occitadys will supervise the regional coordination of these 13 correspondents.

#### First Level: Doctors' Information and Qualifications

This will be coordinated by Occitadys, with the design of a 10-h Continuing Medical Education (CME) program for Doctors. This program will allow Doctors to evaluate reading/spelling/counting abilities through a specifically designed tool, BMT-a (46), and identify attention and motricity warning signs through two parental questionnaires: SNAP IV-26 for ADHD (47) and DCDQ FE 5-15 for coordination disorders (48). The program will be supported by the local experts in charge of the second-tier response, and will rely on the typical funding for this kind of exercise through the National Agency for Professional Continuous Development (ANDPC: *Agence Nationale pour le Développement Professionnel Continu*). Meetings with Doctors in each *département* will also be organized regarding the new measures to be tested.

#### Second Tier: Testing Multidisciplinary Evaluation and Access to Care Not Previously Covered by Social Security

This is aimed at children aged 6 to 15 with suspected complex disorders, as defined by HAS 2018 (7): diagnostic difficulties, comorbidities, or insufficient response from the first care tier. Referral may follow an initial first-tier evaluation or be direct for complex disorders affecting different individual functions. As noted in HAS, 2018 (7), “*This second level (between the first level: primary care, and the third level: Reference Centers) concerns children whose situation requires multidisciplinary coordination and the expertise of the coordinator and the whole team. Level 2 aims to coordinate between organizations and professionals as close as possible to the child's home and within a reasonable time, providing expertise and specific skills for TSLA care*”.

All of the different evaluations and care are potentially available, and will be assigned according to the needs of the child and the family. The recruitment of level-2 expert MDs in the Occitanie region has been helped by a longstanding policy (since 2007) of University Diplomas in the two main urban centers, Toulouse and Montpellier, allowing the reinforcement of existing multi-disciplinary teams, and promoting the creation of new teams in 7 out of the 13 *départements*. Third tier (Reference Centers) in the Occitanie Region are present in Toulouse, Montpellier and Tarbes: their organization will not be modified by the experimentation, but we can expect to achieve shorter waiting lists, via better coordination between the three levels.

The financing for each step of the diagnostic process and care is based on data from a national study from a French Users' Association (21), estimating the hours and level of experience required for each position, confirmed by a medico-economic evaluation from one of the second-tier organizations after five years of experience (JC Semet, personal communication), providing a cost per child figure for multidisciplinary evaluation. The additional costs refer to administrative support and the information system. The detailed funding by task or sequence of acts appears in Table 3.

**Table 3:** Cost by act/sequence during the Experiment “Parcours de Santé TSLA Occitanie”.

**Diagnostic Sequence at the second Tier**	
Medical evaluations including two long appointments and a short-term follow-up visit	€300
Paramedical evaluation: psychomotricity or occupational therapy (each)	€150
Standard psychological assessment: anamnesis, psycho-affective history, cognitive functions	€250
Discretionary second-tier psychological assessment: attention and executive functions	€170
Memory assessment	€150
Coordination of health track, including entry correspondent, administration, multidisciplinary summary, and information systems	€250
**Care sequence**	
One year of re-education/rehabilitation with a psychomotricity specialist or occupational therapist, potentially renewed after the first year: €45 per session for a set of 35 sessions	€1450
10 sessions of psychological care addressing the consequences of NDD (anxiety, depression etc.), or for cognitive remediation: €45 per session	€450
10 sessions of parenting-skill training programs: €45 per session	€450
Comprehensive medical evaluation at the end of the first sequence of 12 months of care	€120

#### Information System

The information system will be based on the national My Health 2022 strategy (*Ma santé 2022*), and its subsection “Accelerating digital conversion in health” (*Feuille de route Accélérer le virage numérique en santé*), for which Regional Health Authorities (ARS) are responsible. The experiment will benefit from the results of the tender by ARS Occitanie, with an industrial partner in charge of providing digital infrastructure adaptated to each local project. Our experimental timeline implies an initial digital tool in April 2021 and fully-operational system in September 2021. Funding comes from a special grant from ARS Occitanie, as a starter for the implementation of the “*Parcours de Santé TSLA Occitanie*”, of up to €150 000 over two years.

### Phases of Implementation and Experimentation

Second-tier organizations will be treated in three phases. The first group can be converted directly to the new funding system; the second group require minor adaptations or are close to opening; and in the third group more work is required to identify professionals who can provide the service.

The first group covers second-tier structures in five *départements*: Aveyron (278,000 inhabitants), Gers (190,000), Haute Pyrénées (229,000), Lot (173,000) and Tarn (384,000), which can accept their first patients once the financial contract between the Social Security, Occitadys, and the supporting organizations is signed, starting on January 1^st^ 2021.

The second group will be rolled out between April and November 2021 in three *départements*: Aude (365,000 inhabitants), Haute Garonne (1,317,000), and Hérault (1,107,000), including the two main regional cities of Montpellier (280,000) and Toulouse (480,000).

The last group covers the rest of the region, including Ariège (152,000), Gard (736,000), Lozère (76,000), Pyrénées Orientales (466,000) and Tarn et Garonne (252,000) at a variety of future dates.

The project will run for two years, with the possibility of a one-year extension depending on the speed of roll-out and participation of the primary-care Doctors who can begin to prescribe at the first level in 2021.

### Authorized Funding

Following the specification accepted in July 2020, the split between regional funding from ARS Occitanie and direct Social Security payments to medical professionals is summarized in Table 4.

**Table 4:** Funding allocated to the Experiment “*Parcours de Santé TSLA Occitanie*”.

**Total budget**	**Year 1**	**Year 2**	**Year 3**	**Total**
Regional Intervention Fund ^*^ (FIR: *fond d'intervention régional*) ARS Occitanie	€223 126	€169 218	€106 743	€499 087
National Fund for innovation in Health ^**^ (FISS: *fond pour l'innovation du système de santé) Caisse Nationale d'Assurance Maladie*	€5 024 550	€7 772 368	€8 151 000	€20 947 918
Total	€5 247 676	€7 941 586	€8 257 743	€21 447 005

* *The FIR will cover the roll-out of the project, through a special grant from the Regional Health Authorities, in addition to the annual grant to Occitadys*.

** *Aimed at organizational innovation, the FISS was created by the 2018 Social Security Financing Law to promote new types of financing and organization, with direct payment to professionals*.

### Evaluation

Evaluation will be mainly external, and designed in collaboration with the different partners, according to a reference guide linked to Art 51 (49). The external evaluation will be designed and carried out by an external contractor from a list of organizations previously selected following public tender: via rotation between contractors, the evaluation will be conducted by REES France. From the reference guide, this evaluation will cover three main areas: operative feasibility, efficiency, and the reproducibility and transferability of the experiment at the national level. The analysis will assess the experiment's ability to impact patient care, generate cooperation between the actors involved, and attain full roll-out within the planned timetable, using both qualitative and quantitative methods. The evaluator will analyse the conception of the experiment together with its structure and previous Occitadys interventions, and design a procedure to be submitted to the Social Security. Evaluation will be carried out at two dates: mid-term, to ensure that the ongoing process is adapted to its goals, and an evaluation at the end to be followed by an official report to the French National Assembly.

An internal evaluation will also be conducted by Occitadys and the Associations of Patients and Doctors involved. This will first compare the knowledge of primary-care practitioners before and after the experiment via a large-scale survey. This will cover the regional Representative Union of Health Professionals MD (URPS-ML) in both Occitanie and a control region that is slightly larger in population terms (Auvergne Rhone Alpes (AURA): 7.8 million inhabitants), which already has a network of specialized professionals through a long-established regional network. This will measure the improvement from the Continuing Medical Education (CME) program as part of the experiment. The analysis will cover knowledge regarding all NDD, with a special focus on ADHD, using the same structure of questionnaire as previously used in 1997–1998 for ASD, at the request of the French National Court of Auditors: *Cour des Comptes* (50). ADHD information will also be part of the initial data for a new national analysis planned by HAS in the coming year.

A second evaluation will be conducted by a group of associations representing the families involved. A proposition was made to five of these, all acting at the regional level either directly or through their local branches: FFDys and locally Apedys MP, Association Avenir Dysphasie Midi Pyrenées, DFD 31, DMF 34, and HyperSuper, representing different areas and disorders.

## Discussion

The organization of TSLA care in France relies mainly on two different providers: private practice for the first care level, with the role of GPs and pediatricians, together with re-education that is massively based on private practice, with Social-Security coverage only for speech therapists and orthoptists: all other re-education/rehabilitation is mostly paid directly (and is not reimbursed) by the families. On the contrary, third-tier structures are organized exclusively in the public sector, via direct MoH funding for centers of reference. In between these two, second-tier expertise combines public and private providers, but with considerable geographical differences. Public or associative structures tend to develop following incentives from Regional Health Authorities, while specialized Doctors working in private practice mainly cover large cities or metropolitan areas. The public/private ratio was 74/26 in 2018 and is projected to be 58/42 by 2023 (51). A network of care is also available through associations in different regions in France. The care offered thus differs by area, with public funding available locally through the Regional Intervention Funds (FIR) of the ARS. Therefore, the regional experimentation to set up new instruments in this field of care should take into account the different administrative and economic models.

In general, France is characterized by a large fee-for-service sector for Doctors and those in paramedical professions with a Social-Security tract, and the free market for non-contracted professionals (psychometricians/occupational therapists/psychologists). To date, with the exception of some national and local experiments (in the field of Mental Health), little progress has been made to solve this issue, the goal being directed toward grouped payments for a patient's treatment or for an organization. With the current situation, health inequity remains a major problem for the families that cannot afford care from non-contracted professionals; this is even more the case during the economic crisis following the Covid-19 pandemic.

Like other countries, France has had to implement reforms both to control costs and address service quality. “*Financing systems need to be specifically designed to: provide all people with access to needed health services (including prevention, promotion, treatment and rehabilitation) of sufficient quality to be effective; [and to] ensure that the use of these services does not expose the user to financial hardship*”, as noted in WHO, 2010 (52). There have been a number of steps: since 1996, a Law (LFSS) annually defines the financial position of Social Security, adjusting expenditure according to predicted revenue. This Law is not however binding. An important step toward Universal Health Coverage in France came with the Law of July 1999 defining “*Couverture Médicale Universelle*” (53). In some ways, the experiment that we propose here is a step toward a universal guarantee for a specific service, as sub-classified by Kutsin et al. (54, 55), and so universal health coverage.

An attempt to introduce alternatives to fee-for-service payments appeared in September 2007, with the LFSS 2008 introducing the principle of a pay-for-performance (P4P) system evolving later into a contract based on Public Health Objectives (ROSP) (56–58).

The model chosen in our health track is close to a mixed payment system, adapted to the setting of outpatient care with the vast majority of providers being outside of organizations, with payment per session shared between the different care providers as decided by an expert Doctor. The other bundling options, i.e., the global attribution of resources, have proved inefficient, as in medico-social establishments where the budget would determine what was affordable and could be provided, rather than tailoring provision to patients' needs [adapted from (59)].

**Figure 2:**
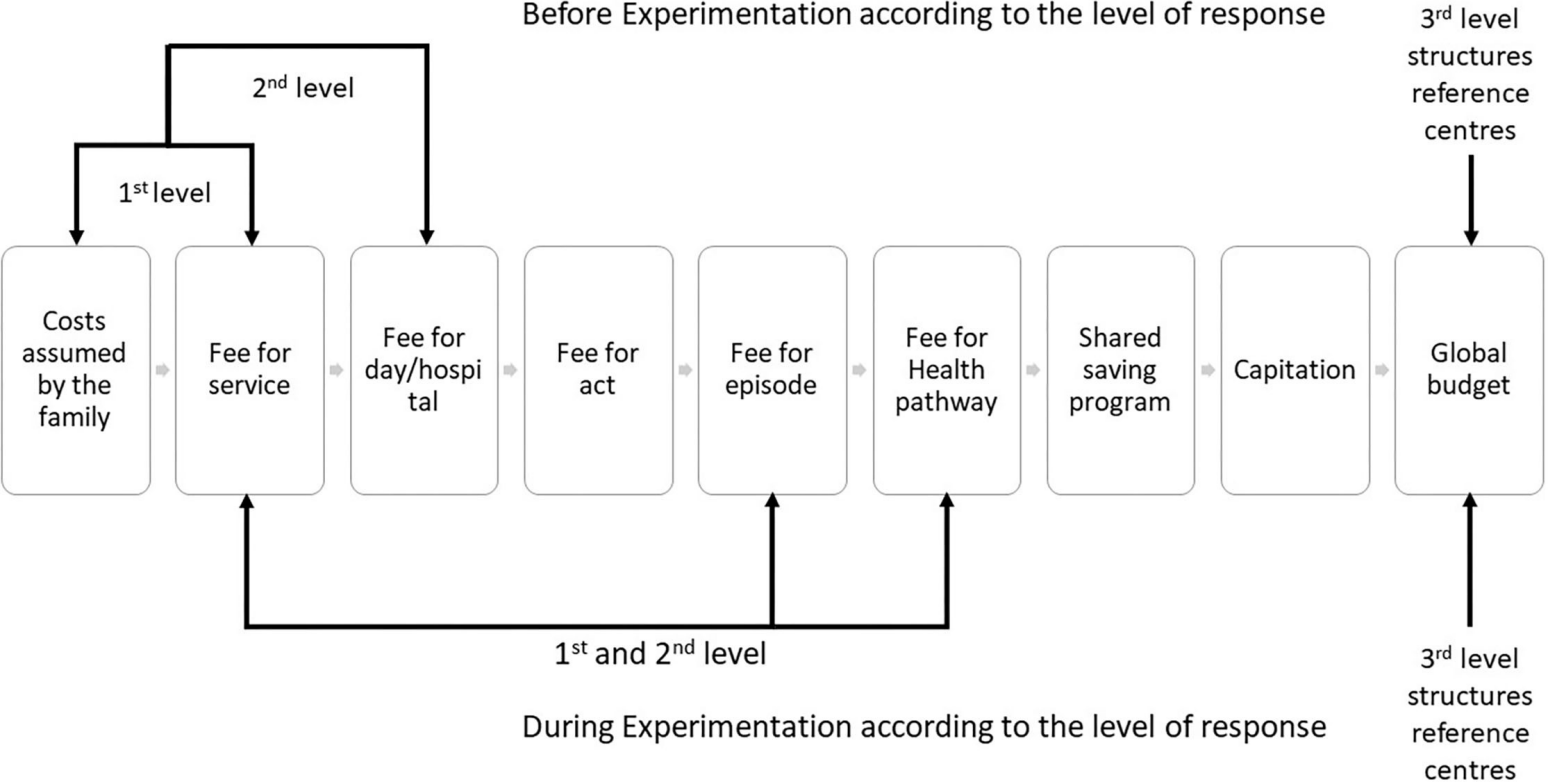
Type of payment procedures in the experimental process: Parcours de Santé TSLA Occitanie (adapted from (60) with permission).

Our experiment addresses a major gap in health coverage, through the design of a health pathway and a combination of different financing strategies that aim to be as close as possible to the local organization, as set out in Figure 2. This strategy was chosen to provide a rapid response for families, without relying on long term-negotiations that would be required with a contract-based approach. Future contracts may however profit from the experiment, especially in establishing the different costs associated with this complex activity.

Our main concern here is universal health coverage via equal access to evaluation and care, producing a more-sustainable system for families by reducing the costs not covered by Social Security. The evaluation of the experiment will not be focused on the cost-effectiveness of the pathway and financing system, as access to diagnosis and care was previously not equal, so there is no adequate baseline for comparison. The evaluation will provide information on the engagement of the funding process, and the gap between predictions and outcomes. However, a recent meta-analysis did not conclude as to a unique model of the strategic purchasing of health-care services that could provide efficient, cost-effective and equal service delivery (58).

The introduction of Article 51 in France has provided a new impetus to experimental projects. These represent a third path, separate from the top-down reforms usually originating from the MoH or Social Security, and the negotiations of contracts between representatives from the medical and paramedical unions. This third option is based on the direct analysis of the situation by professionals and end-users (i.e. representatives of families), with so-called “Medical entrepreneurs” (59) helping design experimental local solutions that can hopefully later be replicated at the national level. At this stage, Occitadys can be considered as such a Medical Entrepreneur, along with professionals (primary-care physicians, specialized NDD Doctors, and paramedical and psychology professionals), care providers (University Hospitals, General Hospitals, private practices, and associations), and patients' and parents' associations, and with the support of local politicians.

The experiment in Occitanie will apply the national expert recommendations from the HAS, 2018, as an evidence-based medicinal program (7), covering as well health care organization but also prevention policies.

In this perspective, Occitadys has also implemented *specific tasks* in the prevention of learning and developmental problems as an essential first stage in the planning of holistic healthcare, and ARS Occitanie has entrusted Occitadys with this role. Prevention and healthcare organization are deeply linked, as reducing patient numbers requiring care will come about via prevention, involving pedagogy based on neurocognitive sciences. To this end, a current regional program is focused on the critical evaluation of the different prevention programs regarding spoken language and reading/spelling learning. A first workshop in April 2021 brought together researchers from a number of French-speaking research centers in order to select the most-accurate/efficient programs as the bases for regional prevention policy in spoken language; the same initiative will take place in 2022 for reading and spelling, and in 2023 for mathematical cognition. A separate strategy will address the improvement of teachers' knowledge of the neurocognitive bases of learning processes, together with the implementation of interventions that have been introduced in a number of countries based on the early identification of difficulties, and the intensification of a pedagogic approach before referral to medical evaluation (61, 62). This synergic approach will ensure an adequate prevention response coupled with satisfactory access to care for children with learning or developmental disorders.

## Abbreviations and Brief Descriptions of the French Organizations Concerned

ARS: “*Agence Régionale de santé*”. The Regional Health Agency, under the control of the MoH, in charge of the regional organization of Health.

MoH: Ministry of Health, “*Ministère des solidarités et de la santé*”. The MOH head changed from Professor Agnès Buzyn to Doctor Olivier Véran, but this did not affect the project. Ministerial delegates cover part of project's domain, with a “*secrétaire d'État en charge de l'enfance et des familles auprès du ministre des Solidarités et de la Santé*” (Childhood and Family State Secretary), and a Minister in charge of Autonomy since July 6^th^ 2020.

A “*Secrétariat d'État chargé des personnes handicapées*” also covers some of the project's concerns, with Mrs Sophie Cluzel as State Secretary attached to the Prime Minister. Last, two delegates are related to our work: Mrs Claire Compagnon, “*déléguée interministérielle à la stratégie nationale pour l'autisme au sein des troubles du neurodéveloppement*”, attached to the Minister in charge for NDD, and Dominique Pon and Laura Létourneau, in charge of the delegation for E-Health, attached to the MoH (“*délégation du numérique en santé*”: DNS).

HAS: “*Haute Autorité de Santé*”. The National Health Authority is a governmental agency, independent from the MoH and Social Security (“*Sécurité Sociale*”), in charge of evaluating health products and technologies, recommending best practices, and measuring and improving quality care and patient safety in healthcare facilities, office-based practice, and social care services and facilities.

*Sécurité Sociale*, alias *Caisse Nationale d'Assurance Maladie*. Social Security in France refers to a set of systems and mainly private institutions whose function is to protect individuals from the consequences of what are generally qualified as “social risks”. There are two main aspects. At the functional level, Social Security assists people when they are faced with costly events or situations. These events are called risks: illness, parenthood, invalidity, death, work accidents, unemployment, occupational illnesses, old-age, and the family. At the institutional level, Social-Security functions are carried out by various organizations, most of them private but with a public-service mission. Only half a dozen national organizations are public (mainly national funds). The staff in these different organizations are therefore, for the most part, not Civil Servants.

Social Security was established by decree on October 19^th^ 1945 by the de Gaulle government. Social Security is a foundation of contemporary French society and economy based on social contributions levied on wages. (Source: Wikipedia; 2020).

## Board of Occitadys

Willig T-N, Henry V, Netter JC, Contis P, Castro-Gutierrez C, Broquère M, Chaix Y, Karsenty C, Leneveu H, Lewandowski C, Leydet J, Paradis-Guennou M, Pupier F, Tardy M, Wioroski M, Bouilhac C, Chaminade S, Charnay M, Chebaiki N, Cicchelero V, Neuhart C, Souksi I, Barry I, Biotteau M, Cojean M, Didillon A, Guitard S, Iannuzzi S, Lafin N, Lubrez L-A, Maillet F, Marizy E, Nesensohn J, Puyjarinet F, Reffuveille I, Taunais A.

## “*Parcours De Santé Tsla Occitanie*” Study Group

Drs Meïer N., Semet J-C, Ruinat P, Karsenty C, Donskoff C, Honegger A, Wallach E, Cadène M-V, Ducos C, Lamonzie PL, Baghdadli A, Purper Ouakil D, Rivier F, Guillard Prudhomme C, Maffre T, Thévenot J, Bensoussan M, Daude H, Kochert F, Branciard L, Groh N, Levrier O.

## Author Contributions

All authors listed have made a substantial, direct, and intellectual contribution to the work and approved it for publication.

## Funding

The conception and initiation of the project was supported through the annual grant from ARS. The medical expenses previously supported by the families will be covered through National Fund for innovation in Health (FISS: *fond pour l'innovation du système de santé) Caisse Nationale d'Assurance Maladie*, without any remaining expenses for the families. Occitadys receives an annual grant from the regional fund for intervention (FIR) from ARS Occitanie, with a contract requiring a detailed annual report of objectives and results, and an external accounting report under French regulations. No other funding is received, especially from private companies, pharmaceutical companies, or any organization that might affect the independence of Occitadys. During project design, Occitadys issued a tender for technical support in the design and writing of the application. Ipso Facto (a cooperative organization) was selected, and AK carried out a mission under a written contract with Occitadys. All these documents are publicly available.

## Conflict of Interest

The authors declare that the research was conducted in the absence of any commercial or financial relationships that could be construed as a potential conflict of interest.

## Publisher's Note

All claims expressed in this article are solely those of the authors and do not necessarily represent those of their affiliated organizations, or those of the publisher, the editors and the reviewers. Any product that may be evaluated in this article, or claim that may be made by its manufacturer, is not guaranteed or endorsed by the publisher.
